# Nutritional content and promotional practices of foods for infants and young children on the spanish market: a cross-sectional product evaluation

**DOI:** 10.1007/s00431-025-06156-y

**Published:** 2025-05-10

**Authors:** Paulina Maria Leszczyńska, Sara de las Heras-Delgado, Sangeetha Shyam, Diane Threapleton, Janet Cade, Jordi Salas-Salvadó, Nancy Babio

**Affiliations:** 1 Universitat Rovira I Virgili, Departament de Bioquímica i Biotecnologia. Grup ANUT-DSM. Unitat de Nutrició Humana. C/Sant Llorenç, 21, 43201 Reus, Spain; 2 Institut d’Investigació Sanitària Pere Virgili (IISPV). Avda, Josep Laporte, 2, 43204 Reus, Spain; 3 Consorcio CIBER, Fisiopatología de la Obesidad y Nutrición (CIBEROBN), Instituto de Salud Carlos III (ISCIII), Av. Monforte de Lemos, 3-5. Pabellón 11. Planta 0, 28029 Madrid, Spain; 4 Nutritional Epidemiology Group, School of Food Science & Nutrition, University of Leeds, Woodhouse Ln, Woodhouse, Leeds, LS2 9 JT UK

**Keywords:** Commercial foods, Infant food, Sugar content, Nutrient profiles

## Abstract

The first 1000 days of life are crucial for health, making it essential that foods for infants and young children (FIYC) meet high nutritional standards, as defined by the World Health Organization's (WHO) Nutrient and Promotion Profile Model (NPPM). There is high reliance on commercially available highly processed FIYC for children under 3 years of age, and a growing market. The aim of this study is to analyse the nutrient profiles of FIYC using the WHO NPPM and determine the proportion of products available in Spain suitable for promotion. Data on 830 FIYC available from brands sold by major retailers in Spain were collected. Product nutrient composition and label information were assessed using the WHO NPPM for the European Region. The processed fruit and vegetables were the most common FIYC category, accounting for 46% of the products surveyed. Overall, only 23% met all NPPM nutritional standards. About 60% of products had excessive sugar content, with 98% of fruit and vegetable purees qualifying for a front-of-pack sugar warning. One in four savoury meals was protein-deficient and one in four of all FIYC was energy-deficient. Only 7% and 2% exceeded the sodium and fat limits, respectively. No product fully met the NPPM promotion recommendations.

*Conclusion*: Nearly 80% of FIYC products in Spain do not meet WHO’s NPPM criteria for nutrient quality, with many high in sugar, low in protein and too watery (low energy). None met the promotional recommendations, highlighting the need for better regulation to improve the nutritional content and appropriate marketing of products for young children.

**Table:** 

**What is Known?** • *The first 1,000 days of life are critical for child development and require optimal nutrition.* • *The WHO European Region developed the Nutrient and Promotion Profile Model (NPPM) to evaluate foods for infants and young children (FIYC)*.
**What is New?** • *This is the first comprehensive study applying the WHO NPPM to 830 FIYC products on the Spanish market.* • *Nearly 80% of products failed to meet the nutritional standards, and none complied with the promotion criteria.*

**Supplementary Information:**

The online version contains supplementary material available at 10.1007/s00431-025-06156-y.

## Introduction

A staggering 4.4 million of children under the age of 5 in Europe suffer from overweight or obesity [1]. The Mediterranean area has the highest rates of overweight, with Spain ranking fourth out of 36 countries in the European region [1]. In an effort to promote healthier diets and prevent childhood obesity, the WHO introduced Resolution WHA 69.9 at the 2016 World Health Assembly, which aimed to “End inappropriate promotion of foods to infants and young children” [2]. To guide Member States in achieving this goal, the WHO commissioned the development of a nutrient profiling tool for commercial baby foods that would follow and improve upon Codex Alimentarius standards and European Commission guidelines. The resulting World Health Organization’s Nutrient and Promotion Profile Model (WHO NPPM) for the European Region assesses both the composition (nutrients and ingredients) and the promotion (labelling and marketing) of commercially available foods for infants and young children (FIYC) [3]. FIYC are defined as a manufactured food or drink, other than breastmilk substitutes, marketed as suitable for the feeding of children under 36 months [3].

Several prior evaluations in Europe, Asia, Africa, North America and Australia have found FIYC to be of poor nutritional quality [4–7] and/or marketed inappropriately [8]. Recent evaluations using the WHO NPPM criteria [3] showed that only 45% of FIYC in the United Kingdom (UK), 25% in Australia and 24% in Malaysia adhered to NPPM nutritional standards [9–11]. This wide variation in the level of compliance to recommended standards makes local evaluations important. More importantly, no product in any of these countries met the promotional requirements recommended in the WHO NPPM. This raises concern as health and nutrition claims create a “halo” effect whereby products appear healthier than they are, appealing to parents and influencing their decisions [8]. An updated evaluation of nutritional quality and promotional practices of FIYC available in Spain was not available. [12] Hence, this study aimed to: 1) compile the nutritional information declared on the packaging of products intended for infants and young children under 36 months; 2) assess compliance with the WHO NPPM criteria; and 3) identify the proportion of products that would require Front-of- Package (FOP) labelling alerts and restricted from marketing and advertising for FIYC in Spain.

## Materials and methods

### Study design

This is a cross-sectional survey of FIYC available in Spain.

### Study food sample

FIYC sold in supermarkets with the highest market share in Spain were surveyed from their physical stores and official websites between June and October 2023. Nine leading supermarkets in Spain were selected based on market share as reported by Kantar and Statista [13, 14] : Mercadona, Carrefour, Lidl, Grupo Día, Grupo Eroski, Consum Coop., Alcampo, Corte Inglés, and Aldi [13, 14].

#### Inclusion criteria

FIYC were defined according to the WHO NPPM profile criteria [3]. These included foods recommended for introduction at an age of < 3 years of age or labelled with the words “baby”, “toddler”, “young child”, or synonyms; or had a label with an image of a child who appeared to be < 3 years of age or was being fed with a bottle.

#### Exclusion criteria

Breast-milk substitutes, vitamin and mineral supplements, FIYC that did not have a Spanish-language website or were marketed to children older than 3 years. Duplicate products that differed only in the number of servings contained were also excluded.

### Food sampling and data collection

FIYC were sampled through physical stores as well as retailer and brand websites in Spain. Regional variations were accounted for by combining brand and in-store retail data. Nutritional information, ingredient lists, and packaging images were collected and documented in a spreadsheet. (further details are reported in Supplementary Methods).

### Defining composition profile and promotional strategies according to WHO NPPM criteria

Composition criteria tailored to specific categories, as defined by the WHO NPPM [3] were used to assess the nutritional content of commercially available FIYC. Briefly, they include four main categories: (i) *Prohibited ingredients:* foods with added free sugars or sweeteners, trans-fatty acids, and those classified as confectionery or sweetened/flavoured drinks; (ii) *Restricted ingredients:* for foods in this category, the amount of fruit in meals, cereals, and dairy products should not exceed 5%. This restriction aims to limit excessive fruit content, which could contribute to high sugar intakes; (iii) *Maximum standards:* sets maximum limits for certain nutritional components, including sodium and total fat. It also specifies that snacks should not exceed 50 kcal per serving, and that the contribution of sugar from savoury meals and snacks should not exceed 15% of the total energy; and (iv) *Minimum standards,* which require the protein content of meals and the energy density of all categories to meet category-specific minimum standards.

In addition, promotional information extracted from the packaging of FIYC was also evaluated using the criteria outlined in the WHO NPPM [3]. Briefly, they encompass promotional strategies that protect and promote breastfeeding, recommend the introduction of complementary foods for infants aged 6 months and older, provide appropriate preparation instructions, use product names that accurately reflect their ingredients, and refrain from making inappropriate claims to influence consumer choice. It is important to note that, in line with the WHO NPPM, the term'promotional practices'used in this study refers specifically to promotional elements on product packaging. These include nutrition and health claims, images and text that may influence the purchasing decisions of caregivers. It is important to highlight that this definition does not include broader retail marketing strategies such as price discounts, product placement or in-store advertising.

### Data analysis

An analysis was conducted using a pre-designed Microsoft Excel spreadsheet for the WHO NPPM product assessment developed by the Nutritional Epidemiology Group at the University of Leeds [15]. Each product was classified according to predefined food categories and subcategories. To assess the product composition and promotional strategies, required data were collected from the FIYC labels and information on the product or its packaging. Further details are included in Supplementary Methods.

The completed spreadsheet was then uploaded to the official WHO NPPM website (https://babyfoodnppm.org/) and the platform automatically scored each product against the WHO NPPM requirements for content (nutrients and ingredients) and promotion (labelling and marketing), according to the product category. For example, fruit-based products and dairy foods that exceed sugar thresholds of 30% and 40% of total energy respectively, would be required to display front-of-pack labels to highlight high sugar content, whereas savoury meals and snacks are required to have a maximum of 15% energy from sugar. An autogenerated report of the'pass'/'fail'results of the products was subsequently downloaded from the website for analysis.

Descriptive analysis in line with the recommended reporting when utilising the WHO NPPM to evaluate FIYC (frequencies, percentages, average, as appropriate for the categorical and continuous variables) were performed using STATA version 15 (StataCorp, College Station, Texas). To analyse the set of commercial baby foods according to the WHO (2022) NPPM, we used Stata code provided by the University of Leeds [16].

## Results

### Descriptive results

In total, 68 FIYC brands were identified through an initial search of supermarket websites. Of these, 27 breast-milk substitutes brands were excluded. In total, 830 products from 42 brands were included (Supplemental Table 1). As the WHO NPPM considers “Drinks” and “Confectionery” to be inappropriate for infants and young children, the 29 products (3.5% of the total number of products, confectionery = 8 and drinks = 21) available in the infant and baby food section of supermarkets, corresponding to these two categories automatically failed the NPPM. Thus, 801 FIYC were included in the final analysis (Supplemental Fig. 1). Despite being excluded from detailed NPPM assessment, nutritional profile of drinks and confectionery are shown in Supplemental Table 2. The fruit juices were marketed for infants under 4 months of age and the tea powders were advertised as suitable for consumption from 2 months of age. The surveyed FIYC according to the WHO NPPM categories and subcategories are shown in Supplemental Table 3.

Processed fruits and vegetables were the most common category of commercially produced FIYC, accounting for 46% of the 801 products included in this evaluation. Within this category, fruit purees predominated (99%). Savoury meals accounted for 25% of the FIYC, with pureed versions being more common. The cereals category accounted for 17%, with dry cereals without milk as an ingredient being the most common (94%). Snacks (8%) and dairy products (5%) were the categories with the fewest products.

### NPPM nutritional evaluation

**Fig. 1:**
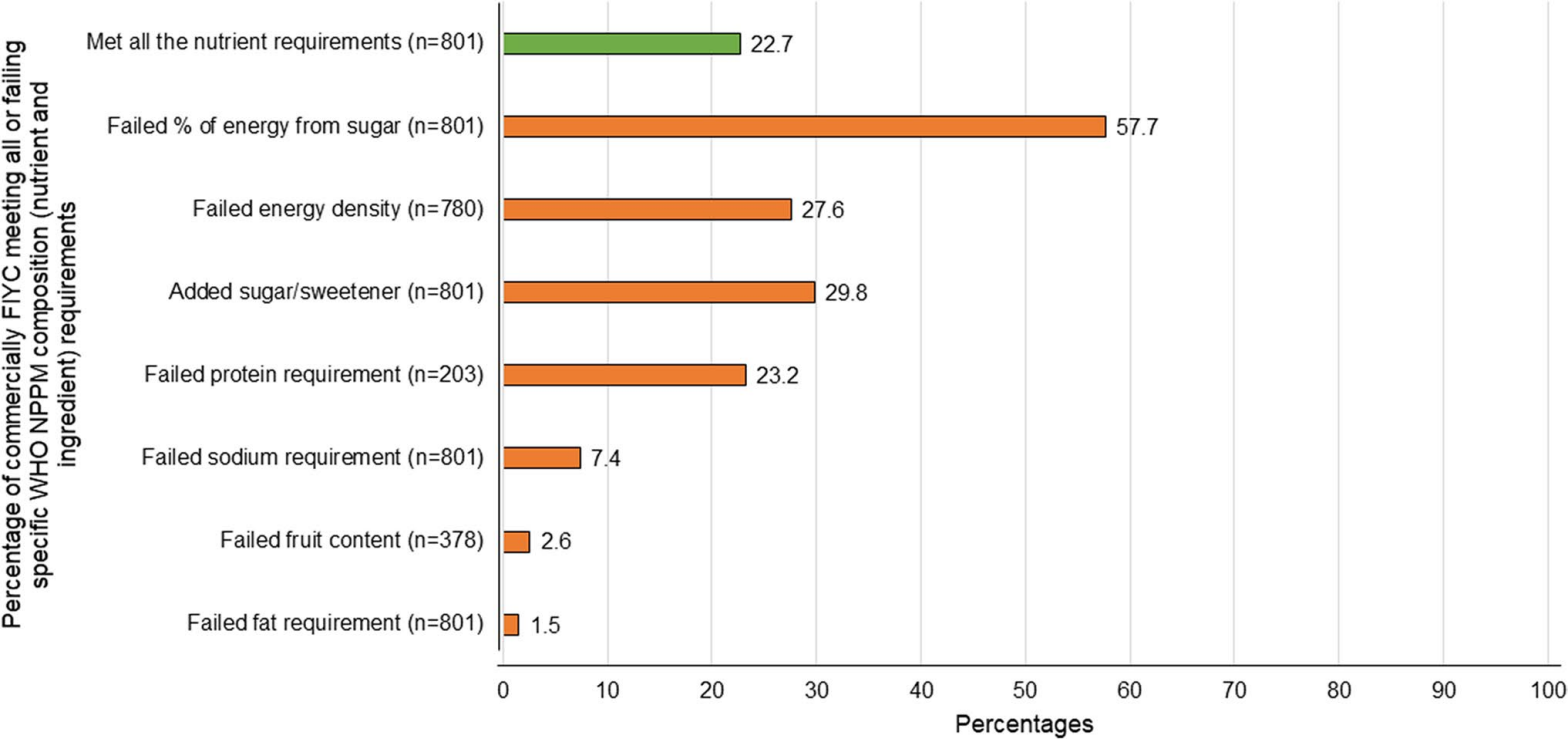
Percentage of commercially available FIYC in Spain failing specific WHO NPPM composition (nutrient and ingredient) requirements. Figure legend: *n* = *Number of eligible products for each requirement Met energy density requirement:* ≥ *60 kcal/100 kcal for cereals, dairy, fruit and vegetable purees, meals;* ≤ *50 kcal per serving size for snacks. Met energy from sugar requirement:* ≤ *15% for meals and snacks;* ≤ *30% for cereals, fruit and vegetable purees;* ≤ *40% for dairy. Met fruit content requirement:* ≤ *10% of dry weight for cereals;* ≤ *5% or* ≤ *2% dry of weight for dairy and savoury meals; no added fruit for vegetable purees. Met protein content requirement:* ≤ *5.5 g/100 kcal for cereals and snacks that contain high-protein food as ingredient; for savoury meals* ≥ *3/4/7 g/100 kcal and* ≥ *8%/10%/40% by weight of the total product depending on the placement of protein source in the product name. Met fat requirement:* ≤ *4.5 g/100 kcal for cereals prepared w/water, dairy, processed fruits and vegetables, snacks and meals without cheese or protein source named first;* ≤ *3.3 g/100 kcal for cereals prepared w/milk;* ≤ *6 g/100 kcal for meals with cheese or protein source named first. Met sodium requirement:* ≤ *50/100 kcal; exceptionally* ≤ *100 mg/100 kcal in meals if cheese named*

Compliance with NPPM nutritional composition requirements (nutrients and ingredients) of commercially produced FIYC available in Spain are shown in Fig. 1 and Table 1. Of the 801 products analysed, 23% (*n* = 182) met all NPPM nutritional requirements (Fig. 1). Compliance to these recommendations varied by FIYC product category: dry cereals: 60%; savoury meals: 39%; snacks: 27%; dairy products: 3% and fruit purees: 1%.

**Table 1:** WHO NPPM composition (nutrient and ingredient) assessment of commercially produced FIYC available in Spain

Product category Number of eligible products for each requirement	*n*	Met all relevant nutrient requirements (*n* = 801)	Failed energy density requirement† (*n* = 798)	Failed energy from sugar requirement‡ (*n* = 801)	Contains added sugar/ sweetener^¶^ (*n* = 801)	Failed fruit content requirement¥ (*n* = 378)	Failed protein content requirement^£^ (*n* = 203)	Failed fat content requirement^&^ (*n* = 801)	Failed sodium content requirement^#^ (*n* = 801)
**Dry cereals (n = 137)**									
1a. Contains milk, prepare with water	2	0.0 (0)	50.0 (1)	0.0 (0)	100 (2)	50.0 (1)	0.0 (0)	0.0 (0)	0.0 (0)
1b. Contains no milk, prepare with water	6	16.7 (1)	33.3 (2)	50.0 (3)	16.7 (1)	16.7 (1)	NA	0.0 (0)	0.0 (0)
1c. Contains no milk, prepare with milk	129	62.8 (81)	6.2 (8)	10.1 (13)	29.5 (38)	5.4 (7)	NA	0.0 (0)	0.8 (1)
**Dairy foods (n = 38)**									
2. Dairy-based foods, desserts, cereals	38	2.6 (1)	2.6 (1)	65.8 (25)	81.6 (31)	0.0 (0)	NA	2.6 (1)	13.2 (5)
**Processed fruit and vegetables (n = 366)**									
3a. Fruit-containing product	363	1.4 (5)	32.2 (117)	98.3 (357)	37.5 (136)	NA	NA	0.0 (0)	2.2 (8)
3b. Vegetable only product	3	0.0 (0)	NA	100 (3)	0.0 (0)	0.0 (0)	NA	0.0 (0)	67.0 (2)
**Savoury meals/meal components (n = 200)**									
4a. Food without protein or cheese named	47	14.9 (7)	42.6 (20)	53.2 (25)	0.0 (0)	2.1 (1)	53.2 (25)	6.4 (3)	29.8 (14)
4b. Food with cheese named but no protein	2	50.0 (1)	50.0 (1)	0.0 (0)	0.0 (0)	0.0 (0)	0.0 (0)	0.0 (0)	0.0 (0)
4c. Food with protein source not named first	122	48.4 (59)	27.9 (34)	13.9 (17)	1.6 (2)	0.0 (0)	9.8 (12)	4.9 (6)	16.4 (20)
4 d. Food with protein source named first	24	37.5 (9)	50.0 (12)	8.3 (2)	0.0 (0)	0.0 (0)	29.2 (7)	0.0 (0)	20.8 (5)
4e. Protein source is only named food	5	40 (2)	20.0 (1)	20.0 (1)	0.0 (0)	0.0 (0)	60.0 (3)	0.0 (0)	20. 0 (1)
**Snacks and finger foods (n = 60)**									
5. Dry or semi-dry snacks and finger foods	60	26.7 (16)	70.0 §(18)	26.7 (16)	48.3 (29)	NA	0.0 (0/1)	3.3 (2)	5 (3)

Data are presented as % (n). § The percentage is calculated including 42 products that showed serving size on the label; and the 18 products that were missing this information are excluded from this analysis

Abbreviation: NA = not applicable for that category

^†^Requirement definition for energy density for category 1a,1b,1c: ≥ 80 kcal/100 g, for category 2/3a/4a/4b/4c/4 d/4e: ≥ 60 kcal/100 g; for category 5: ≤ 50 kcal per suggested serving size (18 missing products are excluded). Not applicable to category 3b

^‡^Requirement definition for energy from sugar for category 1a/1b/1c/3a/3b: ≤ 30% of total energy; for category 2: ≤ 40% of total energy; for category 4a/4b/4c/4 d/4e/5: ≤ 15% of total energy

^¶^The following were considered added sugar/sweetener: sugar, sucrose, dextrose, fructose, glucose, maltose, syrup, nectar, maple, agave, honey, malted barley, malt extract, molasses, fruit juices or concentrated/powdered fruit juice, excluding lemon or lime

^¥^Requirement definition for fruit content for category 1a/1b/1c: ≤ 10% of dry weight; for category 2/4a/4b/4c/4 d/4e/: ≤ 5% of weight or ≤ 2% dry; for category 3b: no added fruit. Not applicable to categories 3a and 5

^£^Requirement for protein content: for category 1a/5, if contains high-protein food as ingredient: ≤ 5.5 g/100 kcal; for category 4a/4b/4c: ≥ 3 g protein/100 kcal and protein named in the product name must be ≥ 8% by weight of the total product; 4 d: total protein ≥ 4 g/100 kcal from the named source and protein named as the first food in the product name must be ≥ 10% by weight of the total product; 4e: total protein ≥ 7 g/100 kcal and protein source mentioned in the product name must be ≥ 40% by weight of the total product. Not appliable to categories 1b/1c/2/3a/3b

^&^Requirement definition for fat for category 1a/1b/2/3a/3b/4a/4c/5: ≤ 4.5 g/100 kcal; for 1c: ≤ 3.3 g/100 kcal; for 4b/4 d/4e: ≤ 6 g/100 kcal

^#^Requirement definition for sodium for category 1a/1b/1c/3a/3b/4a/5: sodium ≤ 50 mg/100 kcal; for category 2/4b/4c/4 d: sodium ≤ 50 mg/100 kcal or ≤ 100 mg/100 kcal if cheese is listed in front- of-pack name

### Energy density and sugar content

Of the 798 products for which an energy density criterium applied (cereals, dairy foods, processed fruit and vegetable, dairy, meals, snacks), 27% did not meet the NPPM thresholds (Fig. 1). Categories with highest proportion of products deficient in energy were processed fruit (fruit purees; 32%, n = 117) and savoury meals (34%, n = 68) (Table 1), with a mean energy content of 52 and 49 kcal per 100 g, respectively (data not shown). Of the 60 snacks and finger foods only 42 featured serving size information on the label, and of those 43% (*n* = 18) exceeded the energy limits (Table 1), with an average of 88 kcal per suggested serving (data not shown).

Overall, 58% (n = 462) of the total study products exceeded recommended limits on the proportion of energy from sugar (Fig. 1). Among the 541 products belonging to dry cereals, dairy foods and processed fruit and vegetable categories, 74% would qualify for a front-of-package (FOP) warning label for exceeding sugar content limits (Table 1). In the processed fruit and vegetables category, almost all products (99%) would require such a warning, with fruit purees averaging 69% of energy from sugar (data not shown).

Regarding added sugars/sweeteners, 30% (*n* = 239) of all analysed products were noncompliant (Fig. 1), particularly those in the fruit puree (57%, n = 136), dairy (13%, *n* = 31) and snack (12%, *n* = 29) categories (Table 1). Of the 378 FIYC that were eligible for a fruit content requirement, only 3% exceeded the limit (Fig. 1).

### Protein, fat, and sodium content

Of the 203 FIYC with protein content requirements, 23% failed to meet the NPPM thresholds (Fig. 1), with savoury meals without a named protein source showing the highest proportion of protein deficient products (Table 1). In terms of fat requirements, the analysis of 801 products showed a high level of compliance, with only 2% exceeding the maximum limits. All dry cereals and processed fruits and vegetables were within the required fat range. Out of the 801 products analysed for sodium content, 7% did not comply with the NPPM (Fig. 1). Savoury meals was the category with the most products that failed the sodium requirement (20%, n = 40). Non-pureed savoury meals aimed at children over 12 months of age were even more likely to exceed sodium limits, with 50% failing and averaging 145 mg of sodium per 100 cal (data not shown).

## NPPM promotional evaluation

**Fig. 2:**
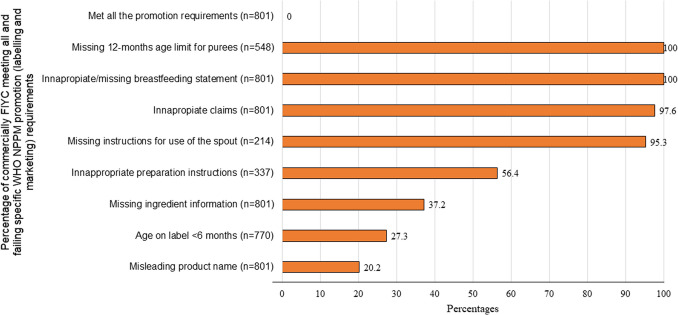
Percentage of commercially available FIYC in Spain meeting all and failing specific WHO NPPM promotion (labelling and marketing) requirements. *n* = *Number of eligible products for each requirement*

The results of the WHO NPPM promotion (labelling and marketing) assessment of commercially produced FIYC available in Spain is shown in Table 2 and Fig. 2. Among the 801 FIYC analysed, none met all promotion requirements (Fig. 2).

**Table 2:** Failure rates in WHO NPPM promotion (labelling and marketing) assessment of commercially produced FIYC available in Spain

Product category Number of eligible products	*n*	Age on label < 6 months (*n* = 801)	12-months age limit for purees (*n* = 566)	Missing instructions for use of the spout (*n* = 214)	Inappropriate preparation instructions (*n* = 337)	Misleading product name (*n* = 801)	Inappropriateclaims (*n* = 801)	Missing information in the ingredients list (*n* = 801)	Inappropriate or missing breastfeeding statement (*n* = 801)
**Dry cereals (n = 137)**									
1a. Contains milk, prepare with water	2	0.0 (0)	NA	NA	50 (1)	0.0 (0)	100 (2)	0.0 (0)	100 (2)
1b. Contains no milk, prepare with water	6	50.0 (3)	NA	NA	83.3 (5)	0.0 (0)	100 (6)	50.0 (3)	100 (6)
1c. Contains no milk, prepare with milk	129	43.4 (56)	NA	NA	65.9 (85)	14.7 (19)	100 (129)	0.0 (0)	100 (129)
**Dairy foods** **(n = 38)**									
2. Dairy-based foods, desserts, cereals	38	3.0 (1)	NA	100 (14/14) †	NA	15.7 (6)	89.4 (34)	50.0 (19)	100 (38)
**Processed fruit and vegetables** **(n = 366)**									
3a. Fruit-containing product	363	37.1 (126)	100 (363)	95.0 (189/199) †	NA	16.0 (58)	97.0 (352)	22.0 (80)	100 (363)
3b. Vegetable only product	3	67 (2)	100 (3)	0.0 (0)	NA	0.0 (0)	100 (3)	66.7 (2)	100 (3)
**Savoury meals/meal components (n = 200)**									
4a. Food without protein or cheese named	47	23.4 (11)	89.4 (42)	100 (1/1) †	59.6 (28)	21.3 (10)	100 (47)	91.5 (43)	100 (47)
4b. Food with cheese named but no protein	2	0.0 (0)	100 (2)	0.0 (0)	100 (2)	0.0 (0)	100 (2)	100 (2)	100 (2)
4c. Food with protein source not named first	122	4.9 (6)	91.8 (112)	0.0 (0)	43.4 (53)	27.0 (33)	97.5 (119)	94.3 (115)	100 (122)
4 d. Food with protein source named first	24	8.3 (2)	87.5 (21)	0.0 (0)	58.3 (14)	100 (24)	95.8 (23)	100 (24)	100 (24)
4e. Protein source is only named food	5	40.0 (2)	100 (5)	0.0 (0)	40.0 (2)	40.0 (2)	100 (5)	100 (5)	100 (5)
**Snacks and finger foods** **(n = 60)**									
5. Dry or semi-dry snacks and finger foods	60	1.7 (1)	NA	0.0 (0)	NA	16.7 (10)	100 (60)	8.3 (5)	100 (60)

Data are presented as % (n)

Abbreviation: NA = not applicable for that category

^†^The percentage is calculated out of the number of products that had a spout: 14 dairy foods, 199 fruit purees and 1 savoury meals

Out of 801 FIYC displaying the recommended age suitability on packs, 26% failed the NPPM’s lower age range requirement of 6 months (Fig. 2). Dry cereals showed highest relative noncompliance, with 43% (*n* = 59) labelled as being suitable for consumption by infants as young as 4 or 5 months. Fruit purees had the highest absolute number of products inappropriately marketed, as 126 were labelled as suitable for 4-month-olds (Table 2). The NPPM also suggests a maximum age limit of 12 months for pureed foods. Out of 548 pureed FIYC there was a 100% failure rate in meeting upper age limit requirement (Fig. 2).

The NPPM requires that pureed foods in spouted pouches clearly advise against letting infants and young children suck directly from the spout. The analysis identified 214 FIYC featuring a spout and 95% (*n* = 204) of them lacked any cautionary note on the label (Table 2).

The NPPM mandates that foods requiring preparation before consumption must clearly instruct preparation instructions on the packaging and recommend that that any added liquid to prepare the product should not contain added sodium or free sugars. Out of the 337 FIYC analysed (dry cereals and savoury meals that exhibited instructions on their packages), 56% (*n* = 190) failed this requirement (Fig. 2). Cereals were the worst-performing category, with 66% of these products featuring unsuitable instructions (Table 2).

To prevent consumer confusion, the NPPM mandates adherence to rules regarding name labelling and discourages misleading product names emphasising positive nutrients or ingredients. Out of 801 FIYC analysed, 20% (*n* = 162) failed these requirements (Fig. 2). Savoury meals presented the highest noncompliance, with 35% (*n* = 69) of products misleadingly placing the protein source first in the product name, despite it not being the largest ingredient by weight (Table 2).

The NPPM stipulates that FIYC should not feature any nutritional, compositional, health or marketing claims on their packaging. Out of 801 products analysed, 98% (*n* = 782) failed this requirement (Fig. 2). Noncompliance was high across all categories: 100% of dry cereals, 100% of snacks, 98% of savoury meals and 97% of fruit purees (Table 2).

The NPPM requires that the list of ingredients clearly states the percentage of the main ingredients. Incomplete ingredient information was found in 37% of studied products (Fig. 2) including 95% of savoury meals, 50% of dairy products and 22% of processed fruit and vegetables, but only 2% of dry cereals (Table 2). Most of these products failed to declare added water information.

To meet the NPPM breastfeeding protection requirement the FIYC must explicitly state the importance of exclusive breastfeeding up to 6 months and continued breastfeeding up to 2 years. Out of 801 products, not one included such a definitive statement (Table 2 and Fig. 2).

## Discussion

This is the first study to examine both nutritional and promotional profiles of a large sample of FIYC in Spain, using the WHO NPPM. In this Spanish evaluation only 23% met all nutritional criteria, with added free sugars often contributing excessively to energy. These findings align with the 22% compliance rate reported in Portugal [17], but show lower compliance than Malta [18], Philippines [19], Australia [20] the United States [21]. It is possible to ascribe this variation to cultural taste preferences or regulatory mechanisms or a combination of both factors [9, 11].

As seen in Australia, foods for infants < 12 months were more compliant than those for older children [20].

Three out four FIYC in our study, required a FOP high-sugar warning, exceeding the 60% reported in Philippines [19] and the 50% in Africa [7]. In the current evaluation, we excluded juices, drinks, and confectionery which automatically fail the WHO NPPM evaluation. Exclusion of these high sugar categories from our analysis explains the lower prevalence (30%) of added sugars observed in our analysis in comparison to the 44% reported in a previous Spanish pilot evaluation which included the aforementioned product categories [22]. Most FIYC, in this study, specifically those belonging to cereals, dairy, processed fruit categories and meals without protein or cheese subcategory were not compliant with the recommendations for sugar intake. Historically, Spain has witnessed market strategies aligned with consumer preferences for sweet-tasting infant foods with fruit purees consistently having higher sugar content [23], suggesting that sweetness may be an important issue in Spanish product formulation. The European Society for Paediatric Gastroenterology, Hepatology and Nutrition (ESPGHAN) advises no added sugars infants < 24 months < 5% of energy from free sugars for older children [24]. However, the outdated Codex Alimentarius and European Union legislation lack regulations on added sugars and do not require their declaration on labels, allowing sweet-tasting FIYC to dominate the market. In early life, there is an innate preference to sweet and umami tastes. This preference may reflect a biological need to optimize growth with food dense in calories and protein and to protect against ingestion of toxic and poisonous substances usually characterised by a bitter taste [25]. Early food preference established during the weaning shape future taste preferences [26]. Repeated exposure to high- sugar/high-salt and ultra-processed food in early life could translate into higher consumption of ultra-processed food with a similar taste profile in later-life, and a long-term displacement of minimally processed food, leading to excessive body weight gain and cardiometabolic conditions. Sweet vegetables, fruit juices, and dairy-like products with high sugar content reinforce a preference for sweet flavours [27], potentially increasing sugar intake later in life [28] and contributing to health issues like dental caries [29], weight gain [30], non-communicable diseases [31, 32]. Early exposure to diverse tastes can moderate this preference and promote healthier habits [33].

A further significant concern was the high rate of low energy density (watery) cereals, dairy, fruit products and meals aligned with the protein deficiency observed in many meals. In this survey we observed over a quarter of all such products failed to meet the minimum energy requirement of 60 kcal/100 g. This NPPM benchmark was set to conservatively match estimates for breastmilk energy density and thus indicates that many products are not providing adequate nutrition, particularly when introduced before 6 months, when nutrient-rich milk intake will be displaced by low-energy, low-protein and often high sugar purees.

This observation for low energy density aligns with data provided from Malta [18] and the UK (25%)(34) and the 16–39% range reported in a European pilot study [35], likely due to a lack of European Union legislation on minimum energy requirements or water content declaration in baby foods.

None of the FIYC evaluated met all the WHO NPPM promotional criteria, consistent with findings from Southeast Asia [6], and US [21]. The NPPM requires the inclusion of breastfeeding statements that emphasize the importance of exclusive breast feeding for 6 months and continued breastfeeding for 2 years. None in our sample totally complied with this requirement. About one in three FIYC were labelled for infants < 6 months, contradicting WHO recommendations for exclusive breastfeeding. European laws, such as Commission Directive 2006/125/EC permit introduction of complementary foods from 4 months onwards, potentially encouraging. premature introduction of FIYC and displacement of breast milk.

Inappropriate marketing claims were present in almost all FIYC, similar to the findings in the United Kingdom [34] and the United States [21] and exceeding rates reported in Malta [18] and the Philippines [19]. Claims create a"health halo"effect that can mislead caregivers into perceiving nutrient-poor products as healthy [2]. Many products labelled as having"no added sugars"still contained free sugars like fruit puree or powder [36]. Such claims may undermine confidence in homemade foods and influence consumer behaviour towards these energy-dense, nutrient-poor options [37, 38].

A major strength of this study is its large, representative sample of FIYC from Spain's leading retailers, including online and in-store data. However, limitations include reliance on label-reported information instead of laboratory analysis and the inability to assess trans fats due to the lack of mandatory disclosure in Europe. This cross-sectional survey while providing a snapshot of the quality of products available in Spain between June and October 2023, does not account for products that have been discontinued in the market and other newer products that have been introduced later. Future research is needed to investigate any mismatch between nutrient/health claims and the actual product nutrient content.

## Conclusion

Nearly 80% of FIYC products in Spain do not meet WHO’s nutritional standards, with many products containing excessive sugar and lacking essential nutrients such as protein and energy. None of the products met WHO's standards for promotional strategies. In light of the fact that Spain is bound by EU legislation, this evidence demonstrates the urgent need for i) Regulatory action at both EU and local (Spanish) levels could contribute to ensuring compliance with WHO recommendations and protecting young children from excessive exposure to added sugars and other critical nutrients, ii) mandatory rather than solely voluntary, reformulation policies to better align FIYC with child nutrition and health priorities, iii) campaigns to educate parents and caregivers on how to avoid inappropriate commercial products. The introduction of FOP labels such as “high in sugar” or “high in salt” will be useful to aid consumers make informed choices. The widespread problems with product composition and misleading labelling highlight the need for stricter regulatory oversight of formulation and advertising, which will improve the nutritional quality of baby foods, ensure accurate consumer information, promote healthy eating habits early in life and help prevent non-communicable diseases in adulthood.

## Supplementary Information

Below is the link to the electronic supplementary material.

ESM 1 (PNG 45.5 KB)

Supplementary file1 (TIF 113 KB)

Supplementary file2 (DOCX 15.3 KB)

Supplementary file3 (DOCX 172 KB)

Supplementary file4 (DOCX 177 KB)

Supplementary file5 (DOCX 18.0 KB)

Supplementary file6 (DOCX 23.3 KB)

## Data Availability

No datasets were generated or analysed during the current study.

## Abbreviations

FIYC: Food for infant and young children
NPPM: Nutrient and Promotion Profile Model
WHO: World Health Organization
