# Supplementary figures and images for: Nutritional content and promotional practices of foods for infants and young children on the spanish market: a cross-sectional product evaluation
Source: Eur J Pediatr. 2025 May 10;184(6):333. doi: 10.1007/s00431-025-06156-y (PMC12065749; doi:10.1007/s00431-025-06156-y)

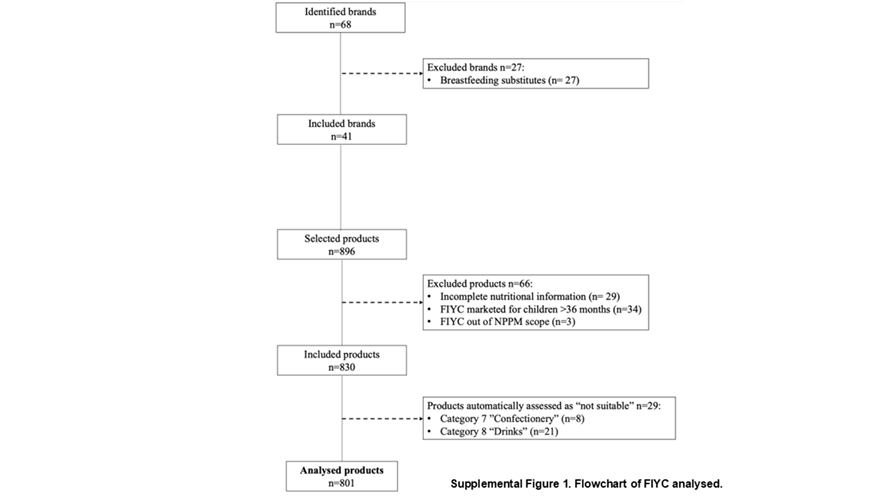

Supplement: Supplementary file 1 — (PNG 45.5 KB) [file 431_2025_6156_Fig3_ESM.png]

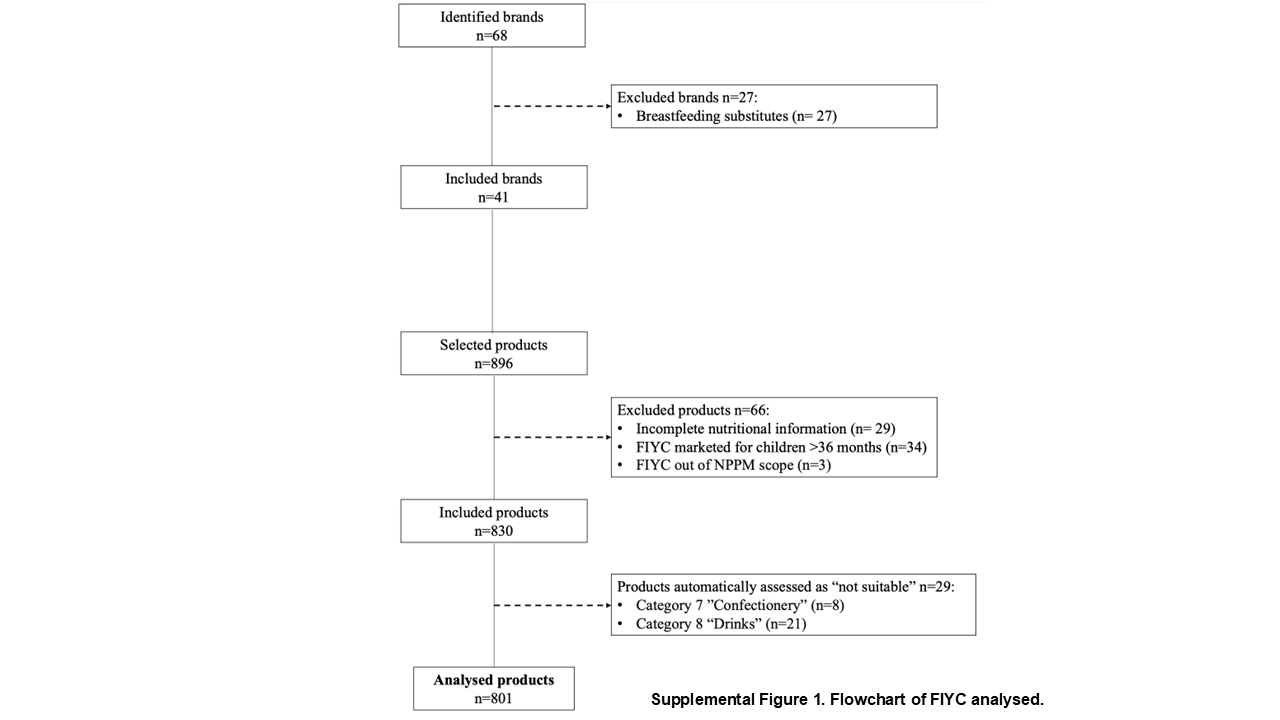

Supplement: Supplementary file 2 — Supplementary file1 (TIF 113 KB) [file 431_2025_6156_MOESM1_ESM.tif]
