# Supplementary material for: Nutritional content and promotional practices of foods for infants and young children on the spanish market: a cross-sectional product evaluation
Source: Eur J Pediatr. 2025 May 10;184(6):333. doi: 10.1007/s00431-025-06156-y (PMC12065749; doi:10.1007/s00431-025-06156-y)
Supplement: Supplementary file 3 — Supplementary file2 (DOCX 15.3 KB) [file 431_2025_6156_MOESM2_ESM.docx]

**Supplemental Table 1: FIYC brands included in this survey.**

| 1 | Alcampo baby |
| --- | --- |
| 2 | Alcampo baby bio |
| 3 | Anela fruits |
| 4 | Aspil |
| 5 | babybio |
| 6 | Be plus (Vicky Foods) |
| 7 | Bebivita |
| 8 | Beech-nut |
| 9 | Blevit |
| 10 | Casa Grande de Xanceda |
| 11 | Damira (Lactalis Nutrition) |
| 12 | Danone |
| 13 | Delikids |
| 14 | Día |
| 15 | Dulcesol |
| 16 | Eroski |
| 17 | Eroski bio |
| 18 | Frullà |
| 19 | Gerber organic (Nestlé) |
| 20 | GoGo Squeez |
| 21 | Good Gout |
| 22 | Hacendado |
| 23 | Hero baby |
| 24 | Hero baby Pedialac |
| 25 | Hero baby SOLO |
| 26 | HiPP biológico |
| 27 | Holle |
| 28 | Libby's |
| 29 | Lupilu |
| 30 | Mi menú |
| 31 | My Carrefour baby bio |
| 32 | Natubé (PedroLuis) |
| 33 | Nestlé |
| 34 | Nutribén (Alter Farmacia))) |
| 35 | Nutricia |
| 36 | Pascual |
| 37 | Planeta bebé (Dia) |
| 38 | Smileat |
| 39 | Snäckids |
| 40 | Vitabio |
| 41 | Yamo |
| 42 | Yogolino (Nestlé) |
