# Supplementary material for: Nutritional content and promotional practices of foods for infants and young children on the spanish market: a cross-sectional product evaluation
Source: Eur J Pediatr. 2025 May 10;184(6):333. doi: 10.1007/s00431-025-06156-y (PMC12065749; doi:10.1007/s00431-025-06156-y)
Supplement: Supplementary file 4 — Supplementary file3 (DOCX 172 KB) [file 431_2025_6156_MOESM3_ESM.docx]

**Supplemental Table 2.** Energy and nutritional content per 100 g of included FIYC (based on label information)

| **Product category** | **Energy**  **(Kcal)** | **Fat**  **(g)** | **Sugar**  **(g)** | **Protein**  **(g)** | **Salt**  **(g)** |
| --- | --- | --- | --- | --- | --- |
| **Confectionery *** (**n=8)** | 314 [291;345] | 1.4 [0.5;5] | 62.5 [37;69] | 1.9 [1.5;4.9] | 0.01 [0.01;0.03] |
| **Drinks*** **(n=21)** | 78 [48;361] | 2.9 [0;3.5] | 6.6 [2.6;81.2] | 2.1 [0;2.8] | 0.07 [0.03;0.13] |

* These categories are not included in the NPPM analysis because they are automatically assessed as not suitable. The eight products classified as confectionery included three bars and five snacks. The 21 drink products included two fruit juices, three sweetened teas and 16 sweetened milk products.

These 29 products account for 3.5% of the total 830 FIYC products surveyed.
