# Supplementary material for: Nutritional content and promotional practices of foods for infants and young children on the spanish market: a cross-sectional product evaluation
Source: Eur J Pediatr. 2025 May 10;184(6):333. doi: 10.1007/s00431-025-06156-y (PMC12065749; doi:10.1007/s00431-025-06156-y)
Supplement: Supplementary file 5 — Supplementary file4 (DOCX 177 KB) [file 431_2025_6156_MOESM4_ESM.docx]

**Supplemental Table 3. Included FIYC according to WHO NPPM categories and subcategories.**

| **Product category** | **Sub-category** | **Examples*** | **n** | % |
| --- | --- | --- | --- | --- |
| **Cereals (n=137)** | Dry cereals containing milk (or milk powder) to be prepared with water | Wheat flour and banana porridge with milk,  Cerelac 5 fruits | 2 | 0.3 |
|  | Dry cereals not containing milk (or milk powder) to be prepared with water | Milk porridge with fruit  Milk porridge with semolina  Organic multicereals | 6 | 0.8 |
|  | Dry cereals not containing milk (or milk powder) to be prepared with milk | Cereals wheat, oats and rice  Cereals with cocoa and quinoa  Superfibre 8 cereals with honey | 129 | 16.1 |
| **Dairy (n=38)** | Dairy product without cheese | Sweetened dairy dessert pouch  Natural yogurt with cow's milk  Custard with cookie | 38 | 4.7 |
|  | Dairy product with cheese | - | 0 | 0.0 |
| **Processed fruits and vegetables (n=366)** | Fruit product, pureed | Apple, banana and strawberry pouch  Apple, peach and pear pouch  100% plant-based coconut, mango and pineapple dessert | 363 | 45.3 |
|  | Fruit product, not pureed | - | 0 | 0.0 |
|  | Vegetable product, pureed | Assorted organic vegetables  Organic carrot  Organic parsnip | 3 | 0.4 |
|  | Vegetable product, not pureed | - | 0 | 0.0 |
| **Savoury meals (n=200)** | Meal, no named protein, pureed | Assorted vegetables and rice  Good night peas corn rice  Vegetables and quinoa menu | 42 | 5.2 |
|  | Meal, no named protein, not pureed | Good night pieces carrot, tomato and polenta (bits)  Mini rigatoni pasta with creamy vegetables sauce  Stars with mediterranean vegetables | 5 | 0.6 |
|  | Meal with cheese in name, pureed | Artichokes, chrivías, pasta and parmesan  Zucchini risotto with goat cheese | 2 | 0.3 |
|  | Meal with cheese in name, not pureed | - | 0 | 0.0 |
|  | Meal, protein not named first, cheese not named, pureed | Vegetables with beef and rice  Vegetables and chicken menu  Vegetables and ham menu | 110 | 13.7 |
|  | Meal, protein not named first, cheese not named, not pureed | Vegetable stew with hake (bits)  Hake casserole with seafood sauce (lumps)  Spaghetti with beef and ham (bits) | 10 | 1.3 |
|  | Meal, protein not named first, cheese named, pureed | Soft zucchini cream with ham and cheese  Vegetables dinner with ham and cheese | 2 | 0.3 |
|  | Meal, protein not named first, cheese named, not pureed | - | 0 | 0.0 |
|  | Meal, protein listed first, cheese not named, pureed | Chicken with vegetables  Chicken with vegetables and rice  Beef with vegetables | 21 | 2.6 |
|  | Meal, protein listed first, cheese not named, not pureed | Chicken with quinoa (chunks)  Salmon with macaroni (chunks)  spaghetti with meat and vegetables (bits) | 3 | 0.4 |
|  | Meal, protein listed first, cheese named, pureed | - | 0 | 0.0 |
|  | Meal, protein listed first, cheese named, not pureed | - | 0 | 0.0 |
|  | Meal, protein only, pureed | Beef planter  Beef stew  100% organic chicken | 5 | 0.6 |
|  | Meal, protein only, not pureed | - | 0 | 0.0 |
| **Snacks and finger foods (n=60)** | Snack, fresh fruit | - | 0 | 0.0 |
|  | Snack, dried fruit | - | 0 | 0.00 |
|  | Snack, no dairy | Cookies for babies  Baked corn aspitos natural  Wheat and oat cereal puffs with organic peanuts | 59 | 7.4 |
|  | Snack, with dairy | Custard cookies | 1 | 0.1 |
| **Ingredients** | - | - | 0 | 0.0 |
| **TOTAL** |  |  | **801** | **100.0** |

*Examples include all products when n in the product sub category was ≤ 3, and randomly picked 3 representative products when n in the product sub category was > 3
