# Supplementary material for: Nutritional content and promotional practices of foods for infants and young children on the spanish market: a cross-sectional product evaluation
Source: Eur J Pediatr. 2025 May 10;184(6):333. doi: 10.1007/s00431-025-06156-y (PMC12065749; doi:10.1007/s00431-025-06156-y)
Supplement: Supplementary file 6 — Supplementary file5 (DOCX 18.0 KB) [file 431_2025_6156_MOESM5_ESM.docx]

**Supplementary Table 4. Missing data information on the criteria**

| Criteria | Number of products with the criteria applicable | Products with missing data | | Remark |
| --- | --- | --- | --- | --- |
|  |  | n | (%) |  |
| Nutritional composition |  |  |  |  |
| Energy density requirement† | 798 | 0 | 0 | Labelling is mandatory. |
| Energy from sugar requirement‡ | 801 | 0 | 0 | Labelling is mandatory |
| Contains added sugar/sweetener | 801 | 0 | 0 | Labelling is mandatory |
| Fruit content requirement¥ | 377 | 0 | 0 | No missing data |
| Protein content requirement^£^ | 203 | 0 | 0 | No missing data |
| Fat content requirement^&^ | 801 | 0 | 0 | Labelling is mandatory |
| Sodium content requirement^#^ | 801 | 0 | 0 | Labelling is mandatory |
| Promotional requirements |  |  |  |  |
| Age on label not <6 months | 801 | 31 | 4 |  |
| Upper age limit of 12 months specified for purees | 548 | 548 | 100 | Missing in all applicable products |
| Instructions for use of the spout included | 214 | 10 | 5 | ‡The percentage is calculated out of the number of products that had a spout: 14 dairy foods, 199 fruit purees and 1 savoury meals. |
| Preparation instructions included | 337 | 5 | 1 |  |
| Product name not misleading | 801 | 0 | 0 | No missing data |
| No inappropriate claims | 801 | NA | NA | Not applicable (NA) as it is criterion that looks for presence or absence of an inappropriate claim. |
| No missing information in the ingredients list | 801 | 0 | 0 | No missing data |
| Appropriate and complete breastfeeding statements | 801 | 801 | 100 | Missing in all products |

†Requirement definition for energy density for category 1a,1b,1c: ≥80 kcal/100 g, for category 2/3a/4a/4b/4c/4d/4e: ≥60 kcal/100 g; for category 5: ≤50 kcal per suggested serving size (18 missing products are excluded). Not applicable to category 3b.   ‡Requirement definition for energy from sugar for category 1a/1b/1c/3a/3b: ≤30% of total energy; for category 2: ≤40% of total energy; for category 4a/4b/4c/4d/4e/5: ≤15% of total energy.   ^¶^The following were considered added sugar/sweetener: sugar, sucrose, dextrose, fructose, glucose, maltose, syrup, nectar, maple, agave, honey, malted barley, malt extract, molasses, fruit juices or concentrated/powdered fruit juice, excluding lemon or lime.  ^¥^Requirement definition for fruit content for category 1a/1b/1c: ≤10% of dry weight; for category 2/4a/4b/4c/4d/4e/: ≤5% of weight or ≤ 2% dry; for category 3b: no added fruit. Not applicable to categories 3a and 5.   ^£^Requirement for protein content: for category 1a/5, if contains high-protein food as ingredient: ≤5.5 g/100 kcal; for category 4a/4b/4c: ≥3 g  protein/100 kcal and protein named in the product name must be ≥8% by weight of the total product; 4d: total protein ≥4 g/100 kcal from the named source and protein named as the first food in the product name must be ≥10% by weight of the total product; 4e: total protein ≥7g/100 kcal and protein source mentioned in the product name must be ≥40% by weight of the total product. Not appliable to categories 1b/1c/2/3a/3b.

^&^Requirement definition for fat for category 1a/1b/2/3a/3b/4a/4c/5: ≤4.5 g/100 kcal; for 1c: ≤3.3 g/100 kcal; for 4b/4d/4e: ≤6 g/100 kcal.  ^#^Requirement definition for sodium for category 1a/1b/1c/3a/3b/4a/5: sodium ≤50 mg/100 kcal; for category 2/4b/4c/4d: sodium ≤50 mg/100 kcal or ≤100 mg/100 kcal if cheese is listed in front- of-pack name.
