# Supplementary material for: Nutritional content and promotional practices of foods for infants and young children on the spanish market: a cross-sectional product evaluation
Source: Eur J Pediatr. 2025 May 10;184(6):333. doi: 10.1007/s00431-025-06156-y (PMC12065749; doi:10.1007/s00431-025-06156-y)
Supplement: Supplementary file 7 — Supplementary file6 (DOCX 23.3 KB) [file 431_2025_6156_MOESM6_ESM.docx]

# Supplemental Methods

# Data collection

The information collection for assessing the nutrient profile and the promotional criteria for the food for infant and young children using the World Health Organization (WHO) Nutrient Profile and Promotion Model (NPPM) is as follows:

**Inclusion criteria:** FIYC were defined according to the WHO NPPM profile criteria (WHO Regional Office for Europe, 2022a). These included foods recommended for introduction at an age of < 3 years of age or labelled with the words “baby”, “toddler”, “young child”, or synonyms; or had a label with an image of a child who appeared to be < 3 years of age or was being fed with a bottle.

**Exclusion criteria:** Breast-milk substitutes, vitamin and mineral supplements, FIYC that did not have a Spanish-language website or were marketed to children older than 3 years. Duplicate products that differed only in the number of servings contained were also excluded.

**Food sampling and data collection**

FIYC were sampled for this project using a two-step process. First, a systematic search of each selected supermarket chain's official Spanish website by two independent reviewers (PML and NB), to identify all FIYC brands offered by the retailer, focusing on the section of the website dedicated to infant food (except two of them, for which a complete search of their website for baby foods were undertaken). The FIYC brands found were systematically documented in a Microsoft Excel spreadsheet, excluding duplicate brands. In the second step, an online search was carried out to identify the official website page of each brand identified and a list of all available FIYC was transcribed into the Excel spreadsheet, along with their respective nutritional information per 100 grams/100 millilitres and ingredient list.

To verify and document the listed FIYC, an on-site survey was carried out in the baby food section of each supermarket between September and October 2023. The two largest retailers from each chain in Reus and Tarragona cities (Catalonia, Spain) were selected. Two separate stores per chain were selected to be surveyed. Each one was surveyed once.

Possible regional variations have been compensated for by the initial survey of each chain's online store, which has deliveries to all regions in Spain. Photographs were systematically taken of both the front and back labels of each FIYC and this information was used to extract the required data. When a FIYC listed in the preliminary spreadsheet was not physically available in the store, a search in the official brand website was undertaken to obtain images of the packaging. If new products were found in the store, they were added to the list.

# Nutrient composition assessment data

To assess product nutrient composition the following information was collected: product weight (grams), dry cereal weight (grams), volume of added liquid for dry cereals (milliliters), energy (kcal), fat (grams), sugar (grams), protein (grams), sodium (milligrams), salt (grams), protein percentage, total fruit percentage, dried fruit percentage, free sugars ('yes' or 'no'), percentage of added water or stock.

# Promotional strategies assessment data

To evaluate promotional strategies, the following data ('yes' or 'no') were also obtained from the product packaging: lower age recommendation (age on label <6 months), upper age recommendation (12-months age limit for purees), missing instructions for use of the spout, inappropriate preparation instructions, misleading product name, inappropriate claims, missing information in the ingredients list, inappropriate or missing breastfeeding statement, serving size for snacks (grams).

**Data Extraction:**

Prior to extraction of data, researchers were trained on data extraction. Random checks were performed by independent members of the team covering approximately 20% of the products, to ensure veracity of the data. The extent of missing data for each criterion is showed in Supplemental Table 4. No imputations were carried out in case of missing data, as if key information was missing the FYIC product was thought to fail that criterion. Missing data information on the criteria are showed in **Supplemental Table 4**.

*Reference: WHO Regional Office for Europe. (2022). Nutrient and promotion profile model: supporting appropriate promotion of food products for infants and young children 6–36 months in the WHO European Region.*
